# 5-[(3,4-Dimethoxy­benzyl)­aminomethyl­ene]-2,2-dimethyl-1,3-dioxane-4,6-dione

**DOI:** 10.1107/S1600536809017413

**Published:** 2009-05-14

**Authors:** Rui Li, Jian-You Shi, Zhen-Yu Ding, Yu-Quan Wei, Jian Ding

**Affiliations:** a State Key Laboratory of Biotherapy, West China Hospital, Sichuan University, Chengdu 610041, People’s Republic of China; b Department of Medicinal Chemistry, West China School of Pharmacy, Sichuan University, Chengdu 610041, People’s Republic of China; c State Key Laboratory of Drug Research, Shanghai Institute of Materia Medica, Chinese Academy of Sciences, Shanghai 201203, People’s Republic of China

## Abstract

The title compound, C_15_H_17_NO_6_, is approximately planar, with dihedral angles of 3.11 (4) and 2.12 (4)° between the connecting amino­methyl­ene unit and the planar part of the dioxane ring, and between the dimethoxy­benzyl ring and the amino­methyl­ene group, respectively. The dioxane ring exhibits a half-boat conformation, in which the C atom between the dioxane O atoms is 0.5471 (8) Å out of the plane. The mol­ecule has an intra­molecular N—H⋯O hydrogen bond which may stabilize the planar conformation. In the crystal, weak inter­molecular C—H⋯O hydrogen-bonding contacts, result in the formation of sheets parallel to the *ab* plane.

## Related literature

For the synthesis of related compounds, see: Cassis *et al.* (1985 ▶). For the synthesis of related anti­tumor precursors, see Ruchelman *et al.* (2003 ▶). For the structure of 5-(amino­methyl­ene)-2,2-dimethyl-1,3-dioxane-4,6-dione, see: da Silva *et al.* (2006 ▶). For Meldrum’s acid, see: Meldrum (1908 ▶).
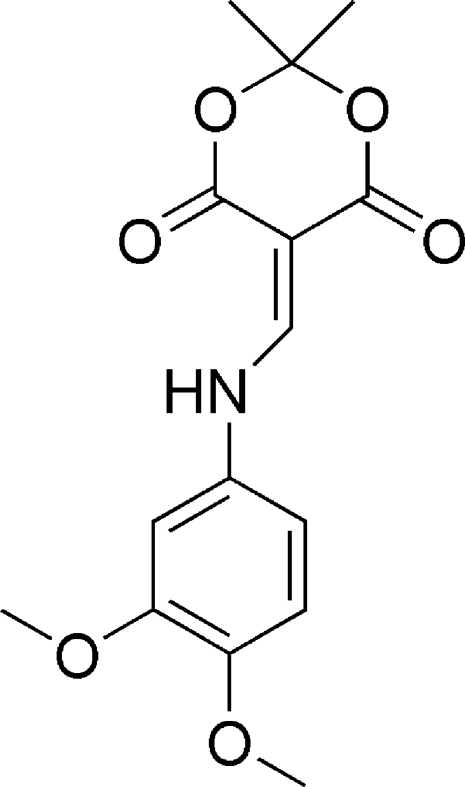


## Experimental

### 

#### Crystal data

C_15_H_17_NO_6_
*M* *_r_* = 307.30
Monoclinic, 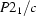

*a* = 6.270 (4) Å
*b* = 12.486 (4) Å
*c* = 19.529 (5) Å
β = 106.31 (3)°
*V* = 1467.3 (11) Å^3^
*Z* = 4
Mo *K*α radiation
μ = 0.11 mm^−1^
*T* = 292 K
0.44 × 0.38 × 0.18 mm

#### Data collection

Enraf–Nonius CAD-4 diffractometer
Absorption correction: none
2852 measured reflections
2657 independent reflections
1675 reflections with *I* > 2σ(*I*)
*R*_int_ = 0.008
3 standard reflections every 150 reflections intensity decay: 1.8%

#### Refinement

*R*[*F* ^2^ > 2σ(*F* ^2^)] = 0.056
*wR*(*F* ^2^) = 0.160
*S* = 1.03
2657 reflections
207 parameters
H atoms treated by a mixture of independent and constrained refinement
Δρ_max_ = 0.21 e Å^−3^
Δρ_min_ = −0.26 e Å^−3^

### 

Data collection: *DIFRAC* (Gabe & White, 1993 ▶); cell refinement: *DIFRAC*; data reduction: *NRCVAX* (Gabe *et al.*, 1989 ▶); program(s) used to solve structure: *SHELXS97* (Sheldrick, 2008 ▶); program(s) used to refine structure: *SHELXL97* (Sheldrick, 2008 ▶); molecular graphics: *ORTEP-3* (Farrugia, 1997 ▶); software used to prepare material for publication: *SHELXL97* and *PLATON* (Spek, 2009 ▶).

## Supplementary Material

Crystal structure: contains datablocks I, global. DOI: 10.1107/S1600536809017413/si2171sup1.cif

Structure factors: contains datablocks I. DOI: 10.1107/S1600536809017413/si2171Isup2.hkl

Additional supplementary materials:  crystallographic information; 3D view; checkCIF report

## Figures and Tables

**Table 1:** Hydrogen-bond geometry (Å, °)

*D*—H⋯*A*	*D*—H	H⋯*A*	*D*⋯*A*	*D*—H⋯*A*
N1—H1*N*⋯O3	0.86 (3)	2.11 (3)	2.744 (3)	130 (2)
C9—H9⋯O4^i^	0.93	2.40	3.309 (4)	164
C15—H15*C*⋯O3^ii^	0.96	2.59	3.528 (4)	166

Symmetry codes: (i) 
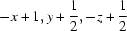
; (ii) 
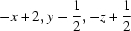
.

**Fig. 1.**
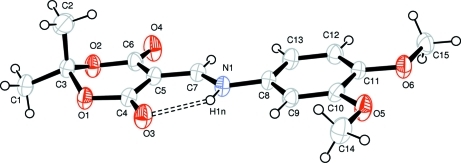
The molecular structure of the title compound, with displacement ellipsoids drawn at the 30% probability level.

**Fig. 2.**
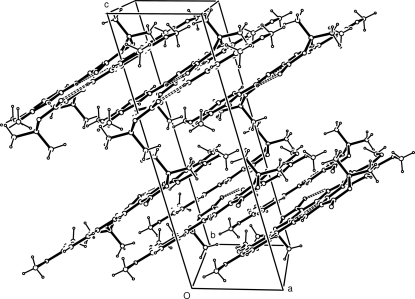
A packing diagram of the title compound showing the layer-like aggregation of the title molecules in the unit cell.

## supplementary crystallographic information

### Comment

The 4(1*H*)quinolone structure plays an extremely important role in the field of pharmaceutical chemistry. These compounds have been used as precursors for anticancer agents, anti-malarial agents and reversible (H+/K+) ATPase inhibitors (Ruchelman *et al.*,2003). 5-arylaminomethylene-2,2-dimethyl-1,3-dioxane-4,6-diones are the key intermediates which can be used to synthesize the 4(1*H*)quinolone derivatives by thermolysis (Cassis *et al.*, 1985).

In the structure of the title molecule (Fig. 1), it is approximately planar with the dihedral angles of 3.11 (4)° and 2.12 (4)° between the connecting aminomethylene unit and the planar part of the dioxane ring, and between the dimethoxybenzyl ring and the aminomethylene group, respectively. Besides, the dioxane ring of the title compound exhibits a half-boat conformation, in which the C atom between the dioxane O atoms is -0.5471 (8) Å out-of-plane.

The intramolecular N—H···O hydrogen bond (Table 1) is stabilizing the planar conformation in the molecule. Intermolecular weak C—H···O hydrogen bonding contacts (Table 1) result in the formation of sheets running parallel to the *a-b* plane in the crystal structure (Fig. 2).

### Experimental

A solution of 2,2-dimethyl-1,3-dioxane-4,6-dione (Meldrum's acid) and methylorthoformate was heated to reflux for two hours and immediately the arylamine was added in an equimolar amount relative to Meldrum's acid. The mixture was heated under reflux for another 5–8 h; single recrystallization from methanol gave the corresponding arylaminomethylene derivative as analytically pure material.

### Refinement

H atoms were positioned geometrically (C—H = 0.93–0.98 Å) and refined using a riding model, with *U*_iso_(H) = 1.2–1.5*U*_eq_(C).

### Crystal data

**Table:**

C_15_H_17_NO_6_	*F*(000) = 648
*M**_r_* = 307.30	*D*_x_ = 1.391 Mg m^−^^3^
Monoclinic, *P*2_1_/*c*	Mo *K*α radiation, λ = 0.71073 Å
*a* = 6.270 (4) Å	Cell parameters from 29 reflections
*b* = 12.486 (4) Å	θ = 4.4–7.7°
*c* = 19.529 (5) Å	µ = 0.11 mm^−^^1^
β = 106.31 (3)°	*T* = 292 K
*V* = 1467.3 (11) Å^3^	Block, yellow
*Z* = 4	0.44 × 0.38 × 0.18 mm

### Data collection

**Table:**

Enraf–Nonius CAD-4 diffractometer	*R*_int_ = 0.008
Radiation source: fine-focus sealed tube	θ_max_ = 25.4°, θ_min_ = 2.0°
graphite	*h* = −7→7
ω/2θ scans	*k* = −15→0
2852 measured reflections	*l* = −23→8
2657 independent reflections	3 standard reflections every 150 reflections
1675 reflections with *I* > 2σ(*I*)	intensity decay: 1.8%

### Refinement

**Table:**

Refinement on *F*^2^	Primary atom site location: structure-invariant direct methods
Least-squares matrix: full	Secondary atom site location: difference Fourier map
*R*[*F*^2^ > 2σ(*F*^2^)] = 0.056	Hydrogen site location: mixed
*wR*(*F*^2^) = 0.160	H atoms treated by a mixture of independent and constrained refinement
*S* = 1.03	*w* = 1/[σ^2^(*F*_o_^2^) + (0.0954*P*)^2^] where *P* = (*F*_o_^2^ + 2*F*_c_^2^)/3
2657 reflections	(Δ/σ)_max_ < 0.001
207 parameters	Δρ_max_ = 0.21 e Å^−^^3^
0 restraints	Δρ_min_ = −0.26 e Å^−^^3^

### Special details

**Table:**

Geometry. All e.s.d.'s (except the e.s.d. in the dihedral angle between two l.s. planes) are estimated using the full covariance matrix. The cell e.s.d.'s are taken into account individually in the estimation of e.s.d.'s in distances, angles and torsion angles; correlations between e.s.d.'s in cell parameters are only used when they are defined by crystal symmetry. An approximate (isotropic) treatment of cell e.s.d.'s is used for estimating e.s.d.'s involving l.s. planes.
Refinement. Refinement of *F*^2^ against ALL reflections. The weighted *R*-factor *wR* and goodness of fit *S* are based on *F*^2^, conventional *R*-factors *R* are based on *F*, with *F* set to zero for negative *F*^2^. The threshold expression of *F*^2^ > σ(*F*^2^) is used only for calculating *R*-factors(gt) *etc*. and is not relevant to the choice of reflections for refinement. *R*-factors based on *F*^2^ are statistically about twice as large as those based on *F*, and *R*- factors based on ALL data will be even larger.

### Fractional atomic coordinates and isotropic or equivalent isotropic displacement parameters (Å^2^)

**Table:**

	*x*	*y*	*z*	*U*_iso_*/*U*_eq_	
O1	0.0589 (3)	0.78796 (12)	0.14968 (8)	0.0456 (5)	
O2	−0.0296 (3)	0.60397 (13)	0.13307 (9)	0.0489 (5)	
O3	0.3798 (3)	0.84242 (14)	0.22145 (9)	0.0522 (5)	
O4	0.2084 (3)	0.47579 (14)	0.18214 (10)	0.0623 (6)	
O5	1.4467 (3)	0.80170 (14)	0.40934 (10)	0.0544 (5)	
O6	1.5852 (3)	0.60587 (14)	0.42558 (9)	0.0503 (5)	
N1	0.7059 (3)	0.69339 (19)	0.27469 (10)	0.0403 (5)	
H1N	0.677 (4)	0.761 (2)	0.2721 (13)	0.046 (8)*	
C1	−0.2969 (4)	0.7333 (2)	0.08019 (14)	0.0523 (7)	
H1A	−0.3797	0.6767	0.0513	0.078*	
H1B	−0.3242	0.7994	0.0540	0.078*	
H1C	−0.3427	0.7404	0.1229	0.078*	
C2	0.0406 (5)	0.7062 (3)	0.03595 (15)	0.0674 (9)	
H2A	0.1966	0.6903	0.0517	0.101*	
H2B	0.0187	0.7749	0.0131	0.101*	
H2C	−0.0343	0.6523	0.0027	0.101*	
C3	−0.0529 (4)	0.70753 (19)	0.09947 (12)	0.0411 (6)	
C4	0.2670 (4)	0.7662 (2)	0.19222 (12)	0.0389 (6)	
C5	0.3347 (4)	0.65633 (19)	0.20110 (11)	0.0374 (6)	
C6	0.1782 (4)	0.5710 (2)	0.17241 (13)	0.0422 (6)	
C7	0.5461 (4)	0.6272 (2)	0.24184 (12)	0.0398 (6)	
H7	0.5778	0.5544	0.2464	0.048*	
C8	0.9280 (4)	0.66471 (19)	0.31291 (11)	0.0374 (6)	
C9	1.0729 (4)	0.74811 (19)	0.34299 (11)	0.0388 (6)	
H9	1.0219	0.8184	0.3384	0.047*	
C10	1.2914 (4)	0.72663 (18)	0.37956 (11)	0.0392 (6)	
C11	1.3659 (4)	0.61937 (18)	0.38805 (11)	0.0370 (6)	
C12	1.2204 (4)	0.5382 (2)	0.35779 (12)	0.0442 (6)	
H12	1.2696	0.4676	0.3626	0.053*	
C13	1.0014 (4)	0.5605 (2)	0.32015 (12)	0.0445 (6)	
H13	0.9050	0.5051	0.3000	0.053*	
C14	1.3812 (5)	0.9112 (2)	0.39922 (15)	0.0588 (8)	
H14A	1.3307	0.9266	0.3491	0.088*	
H14B	1.5056	0.9562	0.4212	0.088*	
H14C	1.2631	0.9245	0.4205	0.088*	
C15	1.6708 (4)	0.4995 (2)	0.43247 (14)	0.0535 (7)	
H15A	1.5973	0.4577	0.4603	0.080*	
H15B	1.8275	0.5012	0.4557	0.080*	
H15C	1.6451	0.4681	0.3860	0.080*	

### Atomic displacement parameters (Å^2^)

**Table:**

	*U*^11^	*U*^22^	*U*^33^	*U*^12^	*U*^13^	*U*^23^
O1	0.0390 (10)	0.0379 (10)	0.0492 (10)	0.0012 (8)	−0.0054 (8)	−0.0002 (7)
O2	0.0380 (10)	0.0398 (10)	0.0585 (11)	−0.0059 (8)	−0.0036 (8)	0.0011 (8)
O3	0.0468 (11)	0.0371 (10)	0.0602 (11)	−0.0067 (8)	−0.0054 (9)	−0.0021 (8)
O4	0.0517 (12)	0.0318 (11)	0.0890 (14)	−0.0007 (8)	−0.0040 (10)	0.0002 (9)
O5	0.0456 (11)	0.0377 (10)	0.0634 (11)	−0.0014 (8)	−0.0120 (8)	−0.0020 (8)
O6	0.0388 (10)	0.0459 (11)	0.0550 (10)	0.0053 (8)	−0.0052 (8)	−0.0005 (8)
N1	0.0345 (12)	0.0390 (13)	0.0417 (11)	0.0005 (9)	0.0012 (9)	0.0041 (9)
C1	0.0384 (15)	0.0603 (18)	0.0493 (15)	−0.0006 (13)	−0.0025 (12)	0.0020 (13)
C2	0.0541 (18)	0.097 (2)	0.0504 (15)	−0.0023 (17)	0.0130 (13)	−0.0022 (16)
C3	0.0356 (13)	0.0432 (14)	0.0392 (12)	−0.0032 (11)	0.0017 (10)	0.0001 (11)
C4	0.0371 (13)	0.0408 (14)	0.0357 (12)	−0.0019 (11)	0.0050 (10)	0.0021 (10)
C5	0.0334 (13)	0.0383 (13)	0.0366 (12)	−0.0023 (10)	0.0034 (10)	0.0010 (10)
C6	0.0338 (14)	0.0417 (15)	0.0471 (14)	−0.0016 (11)	0.0045 (11)	−0.0025 (11)
C7	0.0364 (14)	0.0424 (14)	0.0382 (12)	−0.0001 (11)	0.0063 (11)	0.0013 (10)
C8	0.0329 (13)	0.0404 (14)	0.0346 (12)	0.0006 (11)	0.0024 (9)	0.0052 (10)
C9	0.0410 (14)	0.0334 (13)	0.0377 (12)	0.0042 (11)	0.0038 (10)	0.0017 (10)
C10	0.0413 (14)	0.0378 (14)	0.0329 (11)	−0.0028 (11)	0.0011 (10)	−0.0024 (10)
C11	0.0335 (13)	0.0419 (14)	0.0320 (11)	0.0059 (11)	0.0031 (10)	0.0034 (10)
C12	0.0432 (15)	0.0351 (14)	0.0492 (14)	0.0050 (11)	0.0045 (11)	0.0021 (11)
C13	0.0425 (15)	0.0371 (14)	0.0468 (14)	−0.0042 (11)	0.0011 (11)	0.0043 (11)
C14	0.0604 (18)	0.0389 (15)	0.0644 (17)	−0.0048 (13)	−0.0030 (14)	−0.0025 (13)
C15	0.0455 (16)	0.0508 (16)	0.0572 (15)	0.0149 (13)	0.0030 (12)	0.0028 (13)

### Geometric parameters (Å, °)

**Table:**

O1—C4	1.363 (3)	C2—H2C	0.9600
O1—C3	1.441 (3)	C4—C5	1.432 (3)
O2—C6	1.376 (3)	C5—C7	1.389 (3)
O2—C3	1.439 (3)	C5—C6	1.450 (3)
O3—C4	1.227 (3)	C7—H7	0.9300
O4—C6	1.211 (3)	C8—C13	1.374 (4)
O5—C10	1.359 (3)	C8—C9	1.398 (3)
O5—C14	1.425 (3)	C9—C10	1.382 (3)
O6—C11	1.375 (3)	C9—H9	0.9300
O6—C15	1.424 (3)	C10—C11	1.413 (3)
N1—C7	1.318 (3)	C11—C12	1.380 (3)
N1—C8	1.428 (3)	C12—C13	1.392 (4)
N1—H1N	0.86 (3)	C12—H12	0.9300
C1—C3	1.504 (4)	C13—H13	0.9300
C1—H1A	0.9600	C14—H14A	0.9600
C1—H1B	0.9600	C14—H14B	0.9600
C1—H1C	0.9600	C14—H14C	0.9600
C2—C3	1.513 (4)	C15—H15A	0.9600
C2—H2A	0.9600	C15—H15B	0.9600
C2—H2B	0.9600	C15—H15C	0.9600
			
C4—O1—C3	118.37 (18)	N1—C7—C5	126.0 (2)
C6—O2—C3	118.82 (19)	N1—C7—H7	117.0
C10—O5—C14	117.26 (19)	C5—C7—H7	117.0
C11—O6—C15	117.28 (19)	C13—C8—C9	120.2 (2)
C7—N1—C8	126.4 (2)	C13—C8—N1	122.7 (2)
C7—N1—H1N	117.7 (17)	C9—C8—N1	117.1 (2)
C8—N1—H1N	115.9 (17)	C10—C9—C8	120.4 (2)
C3—C1—H1A	109.5	C10—C9—H9	119.8
C3—C1—H1B	109.5	C8—C9—H9	119.8
H1A—C1—H1B	109.5	O5—C10—C9	125.1 (2)
C3—C1—H1C	109.5	O5—C10—C11	115.5 (2)
H1A—C1—H1C	109.5	C9—C10—C11	119.5 (2)
H1B—C1—H1C	109.5	O6—C11—C12	125.4 (2)
C3—C2—H2A	109.5	O6—C11—C10	115.3 (2)
C3—C2—H2B	109.5	C12—C11—C10	119.3 (2)
H2A—C2—H2B	109.5	C11—C12—C13	121.0 (2)
C3—C2—H2C	109.5	C11—C12—H12	119.5
H2A—C2—H2C	109.5	C13—C12—H12	119.5
H2B—C2—H2C	109.5	C8—C13—C12	119.7 (2)
O2—C3—O1	110.38 (17)	C8—C13—H13	120.1
O2—C3—C1	105.8 (2)	C12—C13—H13	120.1
O1—C3—C1	106.7 (2)	O5—C14—H14A	109.5
O2—C3—C2	110.5 (2)	O5—C14—H14B	109.5
O1—C3—C2	109.8 (2)	H14A—C14—H14B	109.5
C1—C3—C2	113.5 (2)	O5—C14—H14C	109.5
O3—C4—O1	117.1 (2)	H14A—C14—H14C	109.5
O3—C4—C5	125.1 (2)	H14B—C14—H14C	109.5
O1—C4—C5	117.8 (2)	O6—C15—H15A	109.5
C7—C5—C4	121.5 (2)	O6—C15—H15B	109.5
C7—C5—C6	117.6 (2)	H15A—C15—H15B	109.5
C4—C5—C6	120.7 (2)	O6—C15—H15C	109.5
O4—C6—O2	117.6 (2)	H15A—C15—H15C	109.5
O4—C6—C5	127.0 (2)	H15B—C15—H15C	109.5
O2—C6—C5	115.4 (2)		
			
C6—O2—C3—O1	−48.6 (3)	C6—C5—C7—N1	−176.7 (2)
C6—O2—C3—C1	−163.7 (2)	C7—N1—C8—C13	−0.4 (4)
C6—O2—C3—C2	73.1 (3)	C7—N1—C8—C9	179.0 (2)
C4—O1—C3—O2	45.3 (3)	C13—C8—C9—C10	0.7 (4)
C4—O1—C3—C1	159.8 (2)	N1—C8—C9—C10	−178.8 (2)
C4—O1—C3—C2	−76.8 (3)	C14—O5—C10—C9	−2.9 (4)
C3—O1—C4—O3	162.7 (2)	C14—O5—C10—C11	177.2 (2)
C3—O1—C4—C5	−19.1 (3)	C8—C9—C10—O5	178.4 (2)
O3—C4—C5—C7	−3.9 (4)	C8—C9—C10—C11	−1.7 (3)
O1—C4—C5—C7	178.1 (2)	C15—O6—C11—C12	1.5 (3)
O3—C4—C5—C6	171.1 (2)	C15—O6—C11—C10	−177.0 (2)
O1—C4—C5—C6	−6.9 (3)	O5—C10—C11—O6	0.3 (3)
C3—O2—C6—O4	−158.5 (2)	C9—C10—C11—O6	−179.6 (2)
C3—O2—C6—C5	24.7 (3)	O5—C10—C11—C12	−178.3 (2)
C7—C5—C6—O4	2.9 (4)	C9—C10—C11—C12	1.9 (4)
C4—C5—C6—O4	−172.3 (3)	O6—C11—C12—C13	−179.4 (2)
C7—C5—C6—O2	179.3 (2)	C10—C11—C12—C13	−1.0 (4)
C4—C5—C6—O2	4.2 (3)	C9—C8—C13—C12	0.2 (4)
C8—N1—C7—C5	−175.7 (2)	N1—C8—C13—C12	179.6 (2)
C4—C5—C7—N1	−1.6 (4)	C11—C12—C13—C8	0.0 (4)

### Hydrogen-bond geometry (Å, °)

**Table:**

*D*—H···*A*	*D*—H	H···*A*	*D*···*A*	*D*—H···*A*
N1—H1N···O3	0.86 (3)	2.11 (3)	2.744 (3)	130 (2)
C9—H9···O4^i^	0.93	2.40	3.309 (4)	164
C15—H15C···O3^ii^	0.96	2.59	3.528 (4)	166

Symmetry codes: (i) −*x*+1, *y*+1/2, −*z*+1/2; (ii) −*x*+2, *y*−1/2, −*z*+1/2.
